# Rapid detection of predation of *Escherichia coli* O157:H7 and sorting of bacterivorous *Tetrahymena* by flow cytometry

**DOI:** 10.3389/fcimb.2014.00057

**Published:** 2014-05-05

**Authors:** Bradley J. Hernlem, Subbarao V. Ravva, Chester Z. Sarreal

**Affiliations:** ^1^Foodborne Toxin Detection and Prevention Research Unit, Western Regional Research Center, US Department of Agriculture, Agricultural Research ServiceAlbany, CA, USA; ^2^Produce Safety and Microbiology Research Unit, Western Regional Research Center, US Department of Agriculture, Agricultural Research ServiceAlbany, CA, USA

**Keywords:** enteropathogenic *E. coli*, foodborne pathogen, GFP, flow cytometry, protozoa, environmental transport, *Tetrahymena*, *E. coli O157:H7*

## Abstract

Protozoa are known to harbor bacterial pathogens, alter their survival in the environment and make them hypervirulent. Rapid non-culture based detection methods are required to determine the environmental survival and transport of enteric pathogens from point sources such as dairies and feedlots to food crops grown in proximity. Grazing studies were performed on a soil isolate of *Tetrahymena* fed green fluorescent protein (GFP) expressing *Escherichia coli* O157:H7 to determine the suitability of the use of such fluorescent prey bacteria to locate and sort bacterivorous protozoa by flow cytometry. In order to overcome autofluorescence of the target organism and to clearly discern *Tetrahymena* with ingested prey vs. those without, a ratio of prey to host of at least 100:1 was determined to be preferable. Under these conditions, we successfully sorted the two populations using short 5–45 min exposures of the prey and verified the internalization of *E. coli* O157:H7 cells in protozoa by confocal microscopy. This technique can be easily adopted for environmental monitoring of rates of enteric pathogen destruction vs. protection in protozoa.

## Introduction

Protozoa in the environment have been implicated as both potential hosts harboring pathogens (Barker and Brown, 1994) and as agents enhancing pathogen survival and pathogenicity (Rasmussen et al., 2005; Bichai et al., 2008). Presence of the shiga toxin-encoding prophage in *Escherichia coli* O157:H7 (EcO157) is reported to enhance their survival in the food vacuoles of grazing *Tetrahymena pyriformis* (Steinberg and Levin, 2007). The passage of *Salmonella enterica* through and excretion from a soilborne *Tetrahymena* species is reported to convey increased survival of the organism (Brandl et al., 2005). However, protozoan predation was linked to decreases in EcO157 populations in dairy wastewater (Ravva et al., 2010, 2013) but only three ciliate protozoa were isolated in pure culture (Ravva et al., 2010). For these reasons and difficulties in culturing environmental protozoa, it is important to be able to identify protists in the environment which are actively ingesting bacteria and providing a safe-haven for environmental persistence and transport of EcO157 and other human enteric pathogens.

The study of bacterivory by protozoans by flow cytometry presents several challenges, not the least of which is the dynamic range demand on the instrument. In order to discern bacteria from protozoa, they must bear some separately identifiable characteristic. Size is an obvious choice and the different populations can be observed according to their different locations on a side scatter vs. forward scatter plot. Logarithmic scaling is required to overcome the wide variation in size between the bacteria and protozoa. Creative adjustment of signal threshold and detector voltage levels is required when the ratio of protozoa to bacteria is low (Rifa et al., 2002).

However, this gives us no information about protozoa that have ingested bacteria. Generally, this means that the bacteria are labeled with some fluorescent or otherwise identifiable compound. Examples have included chemical staining of the prey bacteria (Gonzalez, 1999; First et al., 2012), *in vivo* expression of fluorescent proteins (Fu et al., 2003) and even bioluminescence (Nelson et al., 2003).

Nonetheless, it is commonly observed that a variety of living cells also exhibit a degree of fluorescence, i.e., auto fluorescence, in the absence of any added fluorescent label. Protozoa typically being much larger than bacteria, they can be weakly auto fluorescent and yet on a cellular basis produce a signal of the same magnitude or greater than a single fluorescently labeled bacterium (First et al., 2012).

These problems are further compounded when studying organisms in environmental samples where the presence of other matter, living and otherwise, makes the identification and sorting of target populations even more difficult. In such cases, the use of nucleic acid staining has proven useful in locating the target organisms in the detritus (Lindström et al., 2002).

In this paper we examine some of these issues using a ciliate protozoan *Tetrahymena* species and pathogenic EcO157 transformed to express green or red fluorescent proteins (GFP or RFP).

## Materials and methods

### Strains and culture

*Tetrahymena* strain SSU was a soil isolate kindly provided by MT Brandl (Brandl et al., 2005) of Produce Safety and Microbiology Research Unit, USDA, ARS, Albany, CA. The protozoa were enriched by the addition of *E. coli* strain DH5-α cells to aqueous soil suspensions, purified and characterized by 18S rRNA gene sequencing (Brandl et al., 2005). The stock culture was grown and maintained in Neff's medium (Neff et al., 1964). Protozoa concentrations were determined (Ravva et al., 2010) using the most-probable number (MPN) method (Blodgett, 2010).

The prey organisms in the grazing experiments were EcO157 strains MM123 and MM127 derived from the Odwalla apple juice outbreak strain RM1484 (Cooley et al., 2006; Ravva et al., 2006, 2010). MM123 and MM127 are spontaneous rifampicin-resistant (100 μg mL^−1^) mutants of RM2315 and RM2318 containing plasmids expressing genes for green fluorescent protein GFP (pWM1029) and red fluorescent protein DsRed (pWM1032, RFP), respectively. The EcO157 strains expressing fluorescent proteins were transformed by WG Miller (Produce Safety and Microbiology Unit) using the plasmids (pWM1029 and pWM1032) he constructed (Miller, Unpublished data). The strains were grown at 37°C on Luria-Bertani (LB) broth supplemented with 50 μg mL^−1^ kanamycin. Cultures were resuspended in 0.01M PBS (pH 7.2) to an optical density of 0.3 at 600 nm to establish a working suspension of 10^8^ cfu mL^−1^ and diluted accordingly as needed. Actual concentrations were monitored by serial dilution and plating on LB agar supplemented with 50 μg mL^−1^ kanamycin. The fluorescent colonies were counted on a UV Transilluminator (Model 3-3000, Fotodyne, Hartland, WI).

### Grazing studies

Grazing experiments were conducted using *Tetrahymena* SSU at a concentration of about 10^4^ protozoan cells mL^−1^ and live prey concentrations ranging from 10^4^ to 10^8^ EcO157 cells mL^−1^ giving prey-predator ratios of 1 to 10,000:1. *Tetrahymena* SSU grown in Neff's medium was amended with 0.01M PBS containing the appropriate concentration of EcO157 cells and incubated at 25 ± 2°C for various intervals during a 4-h period. One-milliliter samples were taken at various intervals for fixation and flow cytometry. Since working with live pathogens require additional safety measures, the uptake of live vs. heat-killed cells at a prey concentration of 10^6^ EcO157 cells mL^−1^ was compared initially at various intervals during a 90-min period. Samples for flow cytometry were fixed with 1% formalin for 10 min at room temperature prior to sorting.

### Flow cytometry and sorting

The instrument used was a FACSVantage SE flow cytometer (BD Biosciences, San Jose, CA) with an Enterprise II, water-cooled Argon laser (Coherent, Santa Clara, CA). The green fluorescence of GFP was quantified using a 530/30 nm bandpass filter and a 630/22 nm bandpass filter was used for the red fluorescence of RFP. Sheath fluid was either FACSFlow (BD Biosciences) or preservative free BioSure flow cytometry sheath solution (BioSure, Grass Valley, CA). The minimum useable nozzle diameter was found to be 70 μm with these organisms. Nonetheless, formalin fixation was required to obtain the *Tetrahymena* intact after sorting. EcO157 cells were inactivated during the fixation process.

### Confocal imaging

Internalization of GFP-EcO157 (MM123) in *Tetrahymena* was monitored after sorting using a Leica TCS-NT confocal microscope equipped HC PL FLUOTAR 20×/0.50 or 40×/0.70 or 63×/1.2 W PL APO objectives, with argon (488 nm), krypton (568 nm), and He/Ne (633 nm) lasers (Leica Microsystems, Wetzlar, Germany) and using Leica TCS NT Software (v. 2.5) for image analysis and preparation. The BP520/50 emission filter set with 488nm laser excitation was used for visualizing the GFP fluorescence. The GFP fluorescence was assigned a green color in the compiled images.

## Results

We compared both GFP and RFP EcO157 for the grazing studies and found that there was no significant advantage of one over the other. Both strains gave good signal strength but the *Tetrahymena* was observed to autofluoresce with approximately equal intensity to the average single bacterial fluorescence signal in either case (data not shown). Because of this autofluorescence, the uptake of bacteria is preferably several-fold in order to significantly resolve the bacterivorous protists from the non-feeding population.

**Figure 1:**
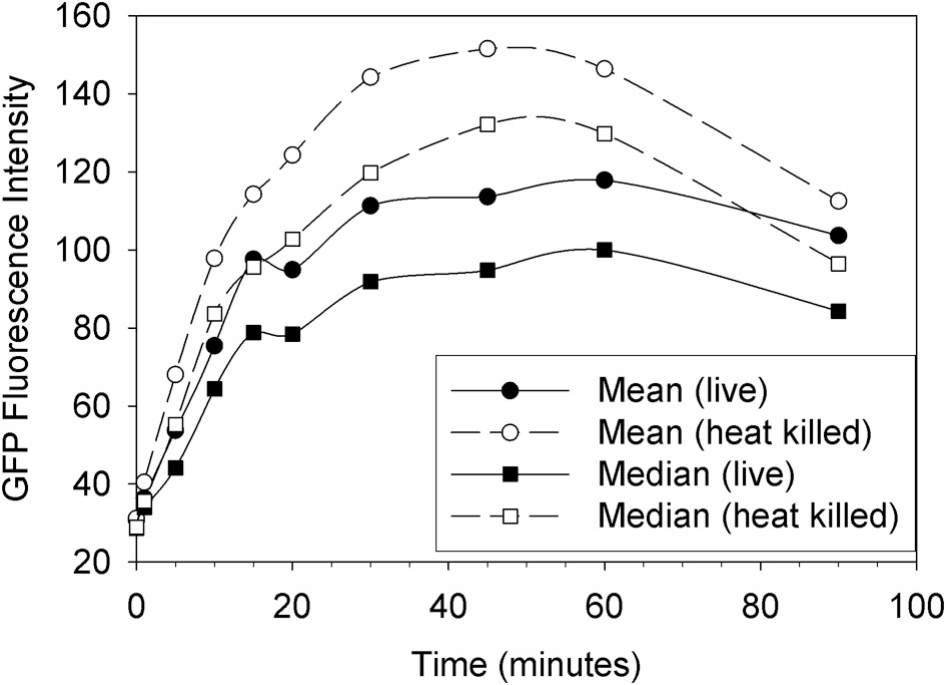
**Uptake of live vs. heat killed cells of GFP-EcO157 by *Tetrahymena* monitored over time as the mean and median green fluorescence intensity**. Open symbols indicate the case where the *E. coli* were heat killed prior to feeding. Initial EcO157 concentration was 10^6^ cells mL^−1^. Predator-prey ratio was 1:100.

The plot in Figure 1 shows a typical time course of fluorescence uptake by *Tetrahymena* feeding on GFP-EcO157 with an initial concentration of 10^6^ mL^−1^. Neither heat nor formalin fixation had an adverse effect on GFP fluorescence of the individual *E. coli* bacteria. Therefore, either method could be used to inactivate the pathogenic bacteria for safety concern when sorting but we chose to use formalin fixation subsequent to feeding so that the bacteria could be fed to *Tetrahymena* live. In any case, the plot shows a rapid uptake of EcO157 whether live or heat killed, the latter yielding somewhat more peak fluorescence internalization.

**Figure 2:**
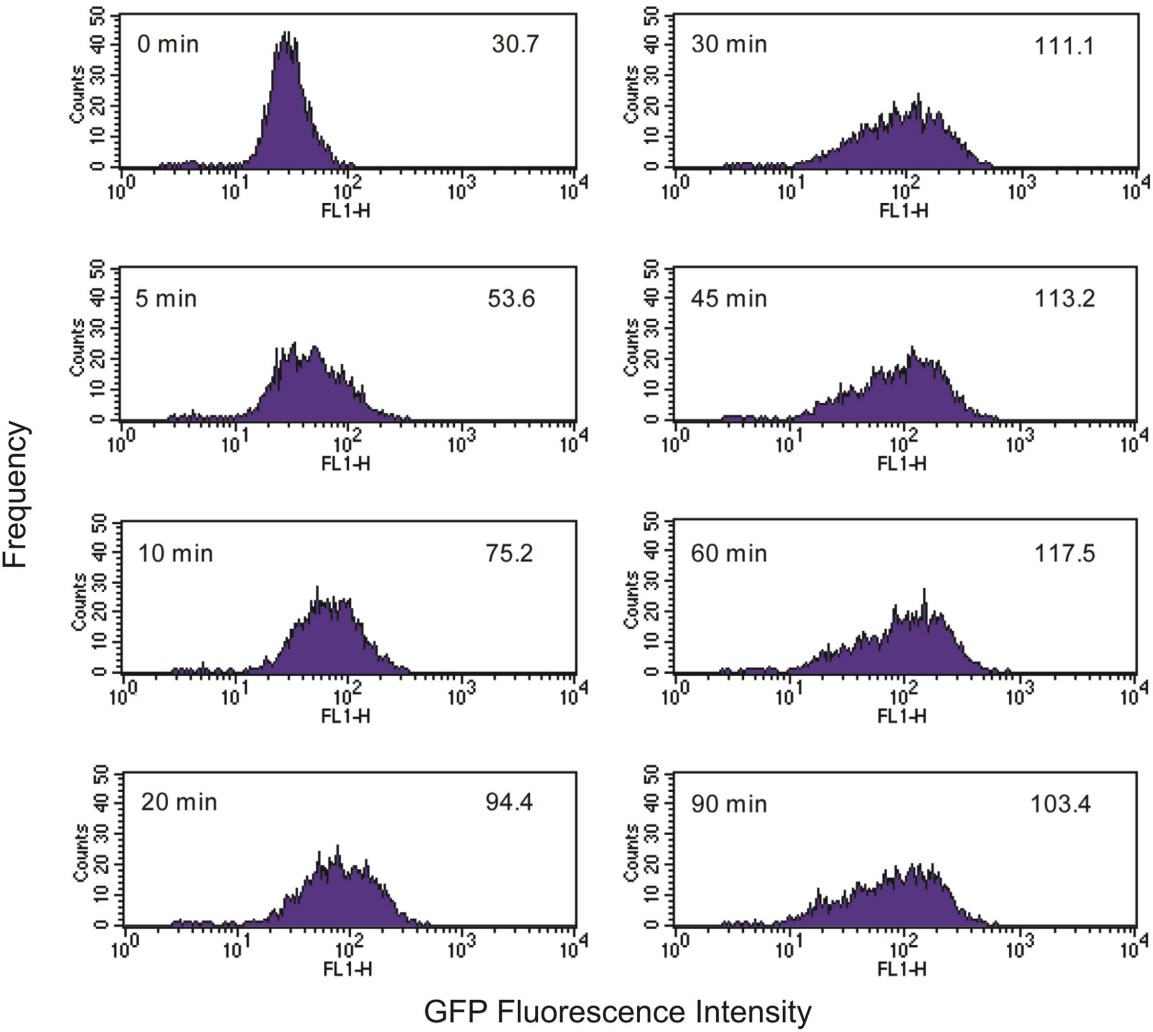
**Time course of green fluorescence uptake of *Tetrahymena* (10^4^ cells mL^−1^) in the presence of an initial concentration of 10^6^ mL^−1^ live GFP-EcO157**. Time and mean fluorescence intensities are indicated in each histogram. Predator-prey ratio was 1:100. See Figure 1 for comparative uptake of heat killed cells.

As *Tetrahymena* feeds on EcO157 cells, there is a broadening of the green fluorescence of the population and a shift to higher average fluorescence (Figure 2). Because neither *Tetrahymena* nor the GFP-EcO157 cells have a narrow fluorescence distribution (fluorescence due to autofluorescence in the former case and GFP in the latter) we do not see discrete increases in fluorescence, i.e. it is not possible from the histograms to discern separate populations according to the numbers of bacteria ingested per cell.

The overall fluorescence of a protozoan cell at any given time is determined by the rate at which it is ingesting fluorescent bacteria, the rate at which it is excreting or digesting fluorescent bacteria, the rate at which the GFP is inactivated inside the *Tetrahymena* as well as the cell's autofluorescence. The ingestion rate is expected to be a function of bacterial concentration and at low enough concentration, the rates of digestion and/or excretion may predominate such that significant fluorescence accumulation is not possible.

**Figure 3:**
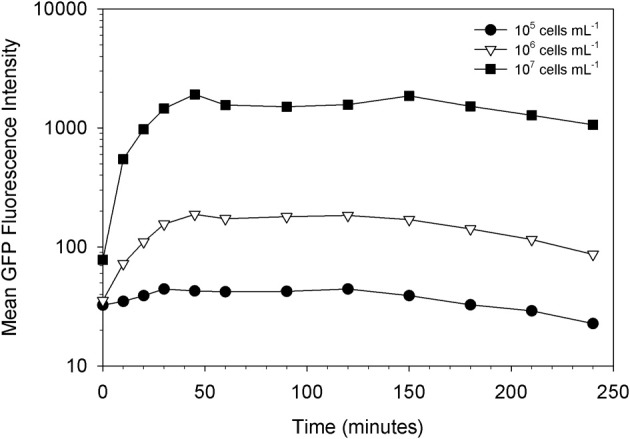
**Influence of prey concentration on uptake of live GFP-EcO157 cells by *Tetrahymena***.

**Figure 4:**
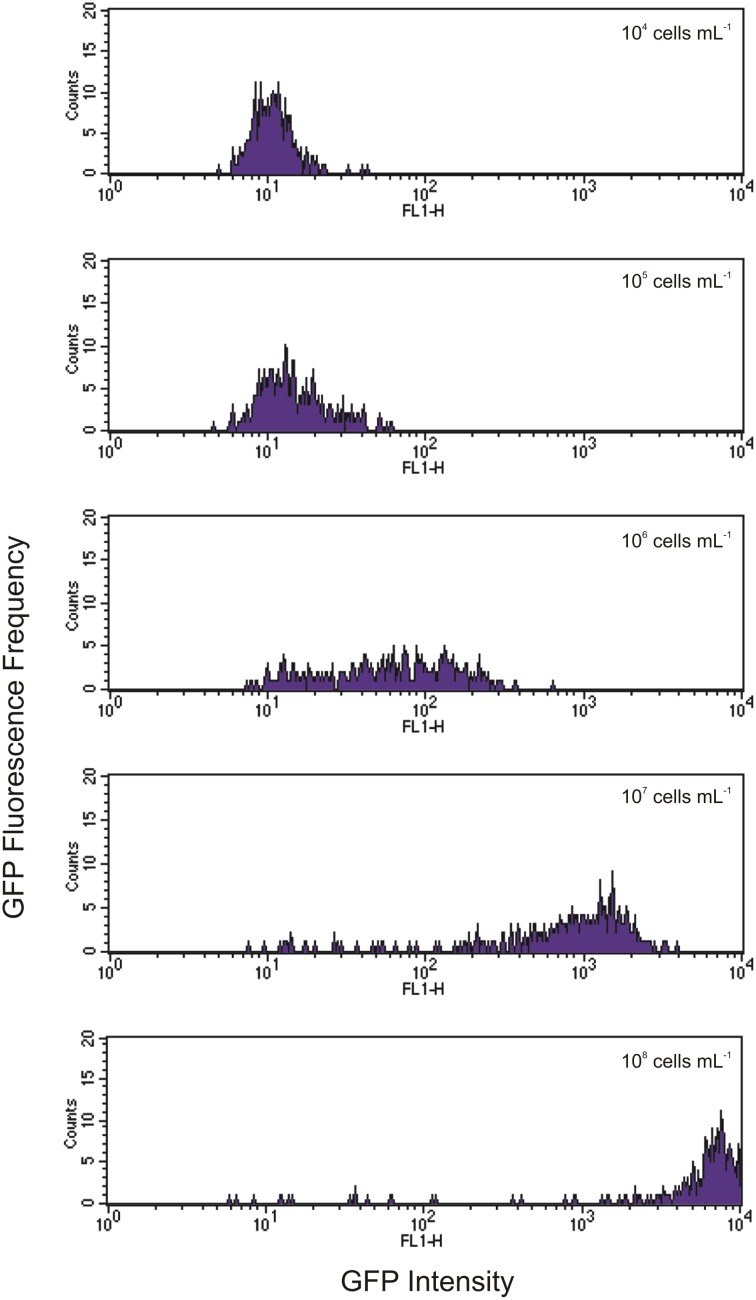
**Histograms showing increasing levels of green fluorescence in *Tetrahymena* after 1 h of feeding with initial concentrations of GFP-EcO157 from 10^4^ cells mL^−1^ (top) to 10^8^ cells mL^−1^ (bottom)**. Each histogram represents a 10-fold increase in initial concentration.

Indeed, we found that the initial concentration of prey is a vital parameter in determining the maximum uptake of fluorescence. This is clearly shown in Figures 3, 4. At the lower initial concentrations of 10^4^ and 10^5^ bacteria mL^−1^, the feeding *Tetrahymena* did not develop enough fluorescence to be clearly discerned from the non-feeding population. On the other hand, as Figure 4 shows, there were very few cells which had no increased fluorescence when the initial bacterial concentrations were 10^7^ and 10^8^ bacteria mL^−1^. The mean fluorescence of the individual *E. coli* and unfed *Tetrahymena* in this experiment were 18 and 16, respectively, on the scale shown.

**Figure 5:**
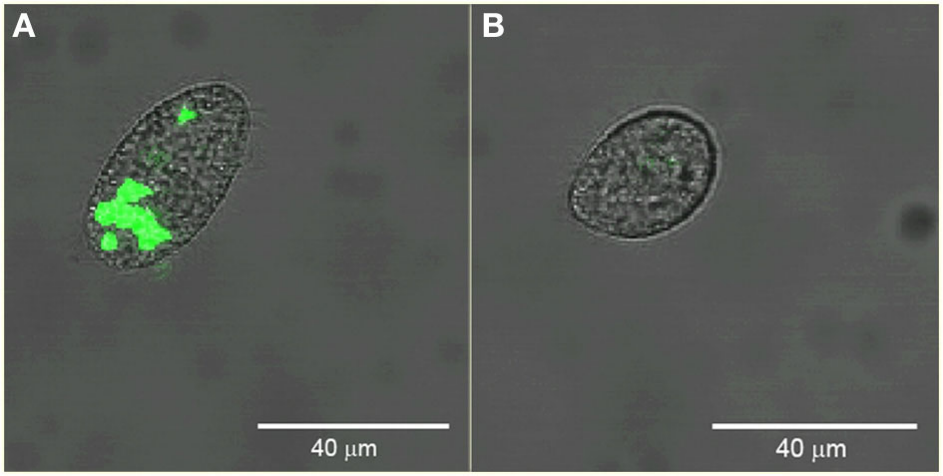
**Confocal images of example *Tetrahymena* sorted from high (A) and low (B) green fluorescence gate regions**. Initial EcO157 concentration was 10^7^ mL^−1^. Cells were fixed after 30 min of grazing. Bars in images are 40 μm in length.

We sorted *Tetrahymena* with high and low levels of green fluorescence. Figure 5 shows confocal images of example cells sorted from these regions verifying the presence and absence of ingested GFP-EcO157 in the two sorted groups. In this example, the main confounding parameters were the high concentration of background bacteria which were 1000-fold more prevalent (10^7^ EcO157 cells: 10^4^ protozoa mL^−1^) than the *Tetrahymena* beside the approximately equal fluorescence intensity of the empty *Tetrahymena* and the single GFP-EcO157 cell. However, even when we added additional debris the feeding *Tetrahymena* could be identified from the background.

## Discussion

The feeding of fluorescent protein labeled prey bacteria is a useful tool to identify and sort bacterivorous protozoa, at least in the case of the ciliate *Tetrahymena*. The ultimate level of fluorescence incorporation is determined by the rates of uptake, excretion and loss due to digestion. It is reported that GFP is unstable and loses fluorescence in the acidic food vacuoles of protozoa even when the bacteria are not completely digested (Parry et al., 2001). We found the fluorescence to be sufficiently persistent in fixed cells to be analyzed and sorted by FACS as well as to be visualized under confocal microscopy. On the other hand, if GFP was persistent in undigested, intact bacteria excreted by *Tetrahymena* (Brandl et al., 2005; Steinberg and Levin, 2007), we should have been able to identify fluorescent vesicles but this was not the case during a 4 h incubation in this study. However, we did observe vesicles filled with GFP-EcO157 (the same strain) expelled from environmental protozoa 2 days after ingestion (Ravva et al., 2010) and internalized GFP-EcO157 cells were detected in protozoa even after 14 days.

One of the hurdles in utilizing this technique is the incorporation of sufficient fluorescence in the protozoa to obtain a separately identifiable population from within environmental samples. This can be overcome by using a sufficient concentration of labeled prey that gives signal significantly higher than the autofluorescence of the target protozoa and other biological and non-biological components. In the environment, the concentration of host and prey would almost certainly be lower than the levels used here. But there seems to be a concentration of prey below which bacterivory is disfavored or at least below which the ingestion rate is too slow relative to excretion and digestion to allow sufficient accumulation of fluorescence to discern. This observation is consistent with the report of Steinberg and Levin that *Tetrahymena* grazing on EcO157 ceases below concentrations of about 10^5^ bacteria mL^−1^ (Steinberg and Levin, 2007) which they attribute to minimum consumption requirement for growth (Watson et al., 1981).

Ingestion rates have been measured by using uniformly fluorescent microspheres (Lavin et al., 1990). Because of the high uniformity of such particles, populations with discrete numbers of ingested particles can clearly be discerned on fluorescence histograms of the protozoa. Although it is not possible with GFP-EcO157 which are not so uniform in size and fluorescence, this method may be used to determine within 5–45 min if environmental protozoa are responsible for significant decreases (Ravva et al., 2010, 2013) of enteric pathogenic bacteria in agricultural environments. Since, isolation and characterization of protozoa in the environment is extremely difficult for use in direct tests to measure the uptake of pathogenic bacteria, flow cytometry appears to be an ideal method in sorting and culturing protozoa based on their relative uptake of fluorescence-labeled organisms. We used flow cytometry earlier to characterize bacteria from urban aerosols (Hernlem and Ravva, 2007). Feasibility studies with environmental samples are needed to determine if protozoa preferentially harbor and transport enteric pathogens from point sources (dairies and feed lots) to fruit and vegetable crops grown in proximity.

### Conflict of interest statement

The authors declare that the research was conducted in the absence of any commercial or financial relationships that could be construed as a potential conflict of interest.
